# Self-evaluated anxiety in the Norwegian population: prevalence and associated factors

**DOI:** 10.1186/s13690-019-0338-0

**Published:** 2019-03-18

**Authors:** Tore Bonsaksen, Trond Heir, Øivind Ekeberg, Tine K. Grimholt, Anners Lerdal, Laila Skogstad, Inger Schou-Bredal

**Affiliations:** 1 Department of Occupational Therapy, Prosthetics and Orthotics, Faculty of Health Sciences, OsloMet, Oslo Metropolitan University, PO Box 4, St. Olavs Plass, 0130 Oslo, Norway; 2 Faculty of Health Studies, VID Specialized University, Sandnes, Norway; 3 Norwegian Center for Violence and Traumatic Stress Studies, Oslo, Norway; 4 Institute of Clinical Medicine, University of Oslo, Oslo, Norway; 5 Division of Mental Health and Addiction, Oslo University Hospital, Oslo, Norway; 6 Department of Behavioural Sciences in Medicine, University of Oslo, Oslo, Norway; 7 Department of Nursing and Health Promotion, Faculty of Health Sciences, OsloMet, Oslo Metropolitan University, Oslo, Norway; 8 Department of General Practice, Institute of Health and Society, Faculty of Medicine, University of Oslo, Oslo, Norway; 9 Department for Patient Safety and Research, Lovisenberg Diakonale Hospital, Oslo, Norway; 10 Department of Nursing Science, Institute of Health and Society, Faculty of Medicine, University of Oslo, Oslo, Norway; 11 Department for Cancer, Oslo University Hospital, Oslo, Norway

**Keywords:** Extraversion, General self-efficacy, Optimism, Neuroticism, Personality, Population survey

## Abstract

**Background:**

Self-evaluations of mental health problems may be a useful complement to diagnostic assessment, but are less frequently used. This study investigated the prevalence of self-evaluated current and lifetime anxiety in the general Norwegian population, and sociodemographic and psychological factors associated with current anxiety.

**Methods:**

A cross-sectional population survey was conducted, using anxiety stated by self-evaluation as outcome. Single and multivariate logistic regression analyses were conducted to examine associations between sociodemographic and psychological variables and anxiety.

**Results:**

One thousand six hundred eighty-four valid responses (34% of the eligible participants) were analysed in this study. One hundred and eleven participants (6.6%) reported current anxiety, while 365 (21.7%) reported lifetime anxiety. Adjusting for sociodemographic and psychological variables, higher age reduced the odds of current anxiety (OR = 0.87, 95% CI = 0.75–0.99), whereas higher levels of neuroticism increased the odds (OR = 2.04, 95% CI = 1.77–2.36).

**Conclusions:**

The study concludes that higher age appears to protect against anxiety, whereas neuroticism appears to increase the odds of experiencing anxiety.

## Introduction

Over years, Norway has received excellent ratings based on a range of indicators, including standard of living and life expectancy in the general population. The population’s education level is high, particularly in the younger age groups – among those aged 25–54 years, 46% has a university or college degree, compared to 33.4% in the European Union [1]. Moreover, the overall unemployment rate is 3.8% [2], in comparison to 8.5% throughout Europe. In spite of these positive indicators, mental health problems are disturbingly common, especially in the urban population segments [3, 4]. Employment is commonly linked with mental health because it provides income, access to social relationships, and the possibility to engage in meaningful activities within a structured environment [5]. Unemployment, on the other hand, has been empirically related to more mental health problems [6–8]. In modern society, formal education functions as the precursor for and gatekeeper of employment, and may in itself add to the person’s resources for sustaining health [9]. In a previous Norwegian study, participants with lower education levels were more likely to have a mental disorder during the last year, compared to those who had higher education [3].

Across the world, anxiety disorders is a category of frequently occurring mental disorders, although prevalence estimates of having any anxiety disorder (last month and during the lifetime) have differed between studies and countries. Steel and co-workers [10] found the global 12-month and lifetime prevalence of anxiety disorders to be 7 and 13%, respectively. However, an insider perspective on mental health might complement the outsider view obtained by diagnostic assessment. Such an insider perspective would emphasize the use of self-report data. To date, no epidemiological studies using self-report data on mental health problems in the general Norwegian population have been published.

Several studies with both clinical and general population samples have investigated anxiety in relationship to gender and personality. There is uniform agreement across studies that women display higher levels of anxiety, compared to men [11]. Similarly, research has shown that women have higher levels of self-reported neuroticism and extraversion [12], and neuroticism in particular has been consistently and strongly associated with anxiety [13]. Studies have also suggested that interactions between personality traits may influence mental health [14].

There are different views regarding the relationship between age and mental health. One view concentrates on ageing as a resource for better psychological coping [15]. In accordance with this view, a large European study found 12-month prevalence rates for any mental disorder and any anxiety disorder to be at 9.6 and 6.4%, respectively [8]. The prevalence of these disease categories tended to decline with age: in the age group 65 years and older, the corresponding prevalence rates were 5.8 and 3.6%. In Norway, the increasing levels of mental health problems among those of younger age, in particular women [16], is a present concern. Recent research on Norwegian students enrolled in higher education found that 19% had serious psychological symptoms [17].

Methodological problems concerned with establishing prevalence rates of mental disorders are varied. For example, while diagnostic categories are dichotomous, symptom levels and perceived burden of disease are presented along a continuum. Considering mental health needs only among those meeting clinically defined thresholds may represent a limitation. Thus, it is important also to estimate the prevalence of self-reported mental health problems, irrespective of clinical diagnosis. Further, the representability of prevalence rates needs consideration. To date, none of the previous Norwegian prevalence studies of mental disorders, conducted in the capital Oslo [3] and in the rural county of Sogn and Fjordane [4], have been considered representative of the country’s population [16]. To represent the entire population of a country the sample needs to reflect the geographical and cultural variations within the country. Both of these methodological concerns were addressed in the present study of anxiety in the Norwegian general population.

### Study aim

This study investigated the point prevalence and lifetime prevalence of self-evaluated anxiety in the Norwegian general population, and sociodemographic and psychological factors associated with current self-evaluated anxiety.

## Method

### Study design and ethics

The Norwegian Population Study (NorPop) is a cross-sectional survey. The collected data reflects a variety of health conditions in the general population and will provide national norm scores related to several questionnaires used for assessing symptoms, attitudes and behavior. No identifying information was collected. The individuals who provided informed consent to participate completed the questionnaires and returned them to the researchers in a sealed envelope. The appropriate ethics committee was consulted and, due to the anonymous data collected, no formal ethical approval was required.

### Sample selection

**Fig. 1:**
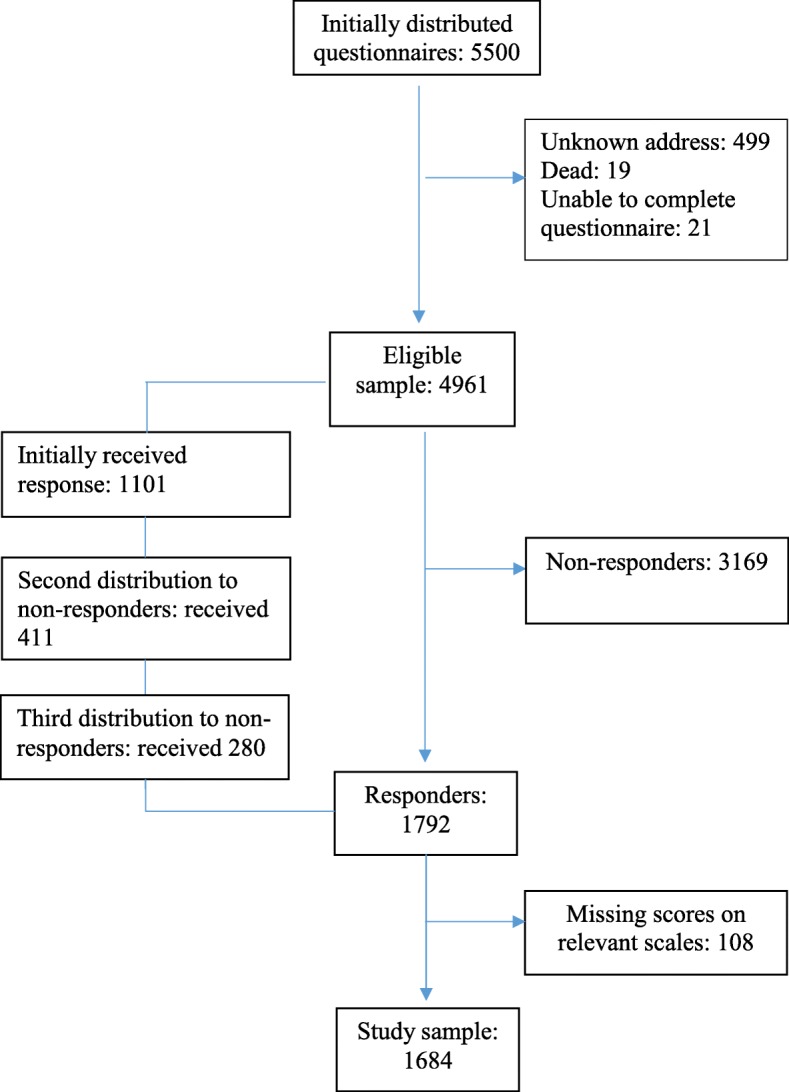
Flowchart showing the inclusion of the participants *in the Norwegian population (NorPop) study, data collected 2015–2016*

A random sample of adult persons (> 18 years of age), stratified by age, gender and geographic region, was approached for possible inclusion in the study. The National Population Register performed the selection. The survey was sent by regular mail to 5500 invited persons along with a letter explaining the purpose and procedures of the study. The flowchart in Fig. 1 displays the recruitment and inclusion process. All data were collected in 2015 and 2016.

### Measures

#### Sociodemographic background

Data regarding age, sex, education, and employment status were collected. The age variable was transformed into age groups: 18–30 years, 31–40 years, 41–50 years, 51–60 years, 61–70 years, and 71 years of age or above. For the inferential analysis, the participants’ actual age was divided by 10 in order to estimate odds change per 10 years increase in age. Formal education level was dichotomized into 12 years’ education or less (coded 0, representing high school or less education) versus more than 12 years’ education (coded 1, representing some level of higher education). Employment status was similarly dichotomized into not working (coded 0) versus working (coded 1). The latter category included persons being employed with paid work, while the former category included persons being retired, unemployed, doing full-time housework, receiving disability benefits, or undergoing education.

#### Anxiety and help seeking

In the present study, we used the phrase: “Below you will find listed some mental health problems. Do you have, or have you had, any of these problems?” One of the listed problems was anxiety. The response alternatives were “no”, “yes previously, but not during the last month” and “yes, during the last month”. Those who confirmed having anxiety in the past (up until the preceding month) or at present were classified as having lifetime anxiety. Further, the respondents were asked: “Have you sought help for your mental health problems”, with the response alternatives “no, not applicable”, “no, but I plan to do so”, or “yes”. Respondents indicating “yes” were then prompted to indicate from whom (general practitioner, psychologist, psychiatrist, district psychiatric center) they had sought help for their mental health problems, currently or previously.

#### General self-efficacy

The *General Self-Efficacy Scale* (GSE) [18] measures self-beliefs related to coping with the demands, tasks, and challenges of life in general. Respondents rate the 10 GSE statements from 1 (not at all true) to 4 (exactly true). Examples of statements are “I can always manage to solve difficult problems if I try hard enough” and “I am certain that I can accomplish my goals”. For the present study, the GSE score was calculated as the mean of all item scores, range between 1 and 4, where higher scores indicate higher general self-efficacy. Factor analysis of the GSE has consistently produced a one-factor solution, which was confirmed in a previous study with the Norwegian general population [19]. Cronbach’s α was 0.92.

#### Optimism

The *Life Orientation Test - Revised* (LOT-R) was used to measure dispositional optimism [20]. The LOT-R consists of 10 self-reported items, where four items are distractors used to disguise the purpose of the measure. Of the remaining six items, three are phrased in an optimistic and three in a pessimistic direction. An example of an optimistic statement is “In uncertain times I usually expect the best”, whereas a pessimistic statement example is “If something can go wrong for me, it will”. The respondents indicated the extent to which they agreed with each of the items on a 5-point scale from 0 (*strongly disagree*) to 4 (*strongly agree*). For the present study, the total LOT-R score was calculated as the mean of the optimism and pessimism item scores, with the pessimism scores inverted. Thus, scores ranged from 0 to 4, with higher scores indicating more optimism. Factor analysis has supported that the LOT-R can be used with a one-factor structure, and Cronbach’s α for the one-factor measure was 0.75 [21].

#### Personality

The *Eysenck Personality Questionnaire* (EPQ) is a self-report questionnaire designed to assess personality traits [22]. In line with previous studies, we used a shortened version of the EPQ, omitting the psychoticism scale. Thus, the EPQ assessed two dimensions of personality: extraversion (degree of liveliness and social orientation) and neuroticism (dispositional worry and nervousness), each assessed with six questions to which the respondent was asked to circle “yes” or “no”. Example statements are “Do you like to meet new people?” (extraversion), and “Are your feelings easily hurt?” (neuroticism). Higher sum scores on each of the scales, both ranging from zero to 6, would indicate higher levels of extraversion and neuroticism, respectively. Factor analysis differentiated between the two underlying dimensions as expected, supporting the validity of the scales. Cronbach’s α was 0.76 for the extraversion scale and 0.77 for the neuroticism scale.

### Statistical analyses

Data were analyzed using SPSS for Windows, version 24. Initial descriptive analyses employed frequencies and percentages for categorical variables (groups categorized by age, gender, education, employment, and presence of anxiety), and means and standard deviations for continuous variables (general self-efficacy, optimism, extraversion, and neuroticism). Single logistic regression analyses were performed, using current anxiety as outcome and each of the independent variables entered separately: age, gender, education level, employment status, GSE mean score, LOT-R mean score, extraversion sum score and neuroticism sum score. Next, the multivariate logistic regression analysis entered all the independent variables into the model. An additional analysis included two interaction terms, neuroticism × extraversion and gender × neuroticism, as independent variables. Finally, the multivariate analyses were performed for two additional outcome variables: current anxiety with help-seeking, and lifetime anxiety. The level of significance was set at *p* <  0.05. Effect sizes in single group comparisons were calculated as Cohen’s *d*, and in the logistic regression analysis as odds ratio (OR).

## Results

### Responders and non-responders

Between responders and non-responders, no significant differences were found with regard to mean age, gender proportions or the distributions of living in rural and urban areas. Among the study participants, 66% were employed, compared to 67% in the general population [2]. Seventeen percent lived alone in both groups. Among the participants, 1.3% were without work and 53% had higher education, compared to 4.4 and 41.0% in the general population [21]. Even though there were somewhat more respondents with higher education than in the general population (53% vs. 41%), we consider the sample to be fairly representative of the Norwegian general population.

### Sample

Altogether, 1792 persons (36.0%) opted to participate in the study. Due to missing data on the scales employed in the current study, 108 responders were excluded, leaving a sample of 1684 participants (34%) for analysis.

### Sample characteristics

The sociodemographic characteristics, state anxiety and scores on the employed scales (GSE, LOT-R, and EPQ) among the participants are shown in Table 1. The mean age of the participants was 52.7 years (*SD* = 16.5 years), with men (*M* = 55.3 years, *SD* = 15.8 years) being older than women (*M* = 50.5 years, *SD* = 16.7 years, *p* <  0.001, *d* = 0.30). Fifty-five percent of the sample had more than 12 years of education, and 61.8% had employment. The point prevalence of anxiety was 6.6% (*n* = 111), the proportions being higher for women (8.2%) than for men (4.7%, *p* <  0.01). The lifetime prevalence was 21.7% (*n* = 365), with proportions being higher for women (25.9%) compared to men (16.9%, *p* <  0.001). Men (*M* = 3.0, *SD* = 0.6) had higher scores than women (*M* = 2.8, *SD* = 0.6) on general self-efficacy (*p* <  0.001). Women scored higher compared to men on extraversion (*M* = 4.1 [*SD* = 1.8] vs. *M* = 3.6 [*SD* = 1.8], *p* <  0.001) and neuroticism (*M* = 2.2 [*SD* = 1.9] vs. *M* = 1.5 [*SD* = 1.7], *p* <  0.001), the latter difference showing a close to medium effect size (Cohen’s *d* = 0.49). Men and women were similar in terms of their scores on optimism (ns.).

**Table 1:** Sociodemographic characteristics of participants (*n* = 1684) in the Norwegian population (NorPop) study, data collected 2015–2016

Characteristics	Sample (*n* = 1684)	Men (*n* = 787)	Women (*n* = 897)	*p*	*d*
Age group	n (%)	n (%)	n (%)		
18–30	203 (12.1)	70 (8.9)	133 (14.8)	< 0.001	
31–40	182 (10.8)	67 (8.5)	115 (12.8)		
41–50	345 (20.5)	145 (18.4)	200 (22.3)		
51–60	340 (20.2)	167 (21.2)	173 (19.3)		
61–70	374 (22.2)	206 (26.2)	168 (18.7)		
71 or above	240 (14.3)	132 (16.8)	108 (12.0)		
Education					
12 years or less	761 (45.2)	368 (46.8)	393 (43.8)	0.23	
More than 12 years	923 (54.8)	419 (53.2)	504 (56.2)		
Employment					
Working	1040 (61.8)	470 (59.7)	570 (63.5)	0.11	
Not working	644 (38.2)	317 (40.3)	327 (36.5)		
Anxiety					
Current anxiety	111 (6.6)	37 (4.7)	74 (8.2)	< 0.01	
Lifetime anxiety	365 (21.7)	133 (16.9)	232 (25.9)	< 0.001	
Past anxiety	254 (15.1)	96 (12.2)	158 (17.6)	< 0.01	
No anxiety	1319 (78.3)	654 (83.1)	665 (74.1)	< 0.001	
Psychological factors	*M (SD)*	*M (SD)*	*M (SD)*		
General self-efficacy (mean)	2.9 (0.6)	3.0 (0.6)	2.8 (0.6)	< 0.001	0.20
Optimism (mean)	2.9 (0.5)	2.9 (0.5)	2.9 (0.5)	0.69	0.02
Extraversion (sum)	3.9 (1.8)	3.6 (1.8)	4.1 (1.8)	< 0.001	0.28
Neuroticism (sum)	1.9 (1.9)	1.5 (1.7)	2.2 (1.9)	< 0.001	0.49

*Note.* ‘Lifetime anxiety’ includes the two categories ‘current anxiety’ and ‘past anxiety’. Statistical tests are *χ*^2^-tests for categorical variables and independent *t*-tests for continuous variables. Effect sizes are calculated as Cohen’s *d*

### Factors associated with anxiety

Table 2 displays the results from the logistic regression analyses. In the unadjusted models, all the independent variables except employment status were significantly associated with the outcome. Current anxiety was associated with lower age, being female, lower education, lower levels of general self-efficacy, optimism, and extraversion, and higher levels of neuroticism. In the multivariate model, controlling for the effects of all independent variables, two variables were significantly associated with the outcome. Each 1-point increase in neuroticism sum score more than doubled the odds of experiencing current anxiety. Gender, education, general self-efficacy, optimism, and extraversion were no longer significantly associated with the outcome. The odds of current anxiety decreased by 13% by each ten-year increase in age. In an additional analysis, we included two interaction terms, gender × neuroticism and neuroticism × extraversion. None of these interaction terms was significantly associated with current anxiety.

**Table 2:** Unadjusted and adjusted logistic regression analyses showing associations between the study variables and current anxiety (*n* = 1684) for participants in the Norwegian population (NorPop) study, data collected 2015–2016

	Unadjusted model			Adjusted model		
Independent variables	OR	*p*	95% CI	OR	*p*	95% CI
Age increase in 10 years	0.80	< 0.001	0.71–0.90	0.87	< 0.05	0.75–0.99
Gender (male vs. female)	1.82	< 0.01	1.21–2.74	1.22	0.42	0.75–1.96
Education (≤12 yrs. vs. > 12 yrs.)	0.63	< 0.05	0.43–0.93	0.82	0.39	0.52–1.29
Working (no vs. yes)	0.77	0.19	0.52–1.14	0.95	0.83	0.59–1.53
General self-efficacy (mean)	0.34	< 0.001	0.26–0.46	0.84	0.37	0.58–1.22
Optimism (mean)	0.30	< 0.001	0.20–0.43	0.75	0.24	0.47–1.21
Extraversion (sum)	0.80	< 0.001	0.72–0.88	0.92	0.17	0.82–1.04
Neuroticism (sum)	2.23	< 0.001	1.95–2.54	2.04	< 0.001	1.77–2.36

*Note.* Reference categories are lower age, male gender, lower education, not working, and lower levels of general self-efficacy, optimism, extraversion, and neuroticism. Adjusted model parameters: Nagelkerke *R*^*2*^ = 0.33, Cox & Snell *R*^*2*^ = 0.13, Model χ^2^ = 218.46, *p* <  0.001. Hosmer Lemeshow: χ^2^ = 6.79, *p* = 0.56

To examine the sensitivity of our analysis, two procedures were performed. First, the logistic regression analysis was re-run restricting the outcome variable to “anxiety with help-seeking” versus all others. Among those with self-reported current anxiety (*n* = 111), four participants did not reveal information related to help seeking for mental complaints. Among the remaining 107 respondents, 70 (65.4%) had sought help. As shown in Table 3, this analysis revealed the same pattern of associations as shown in the main analysis. Neuroticism was significantly associated with higher odds of current anxiety, whereas the association between higher age and lower odds of current anxiety was not statistically significant.

**Table 3:** Adjusted logistic regression analysis showing associations between the study variables and current anxiety with help seeking (*n* = 1684) for participants in the Norwegian population (NorPop) study, data collected 2015–2016

	Adjusted model		
Independent variables	OR	*p*	95% CI
Age increase in 10 years	0.91	0.28	0.77–1.08
Gender (male vs. female)	1.55	0.15	0.86–2.79
Education (≤12 yrs. vs. > 12 yrs.)	0.99	0.96	0.57–1.70
Working (no vs. yes)	0.96	0.88	0.54–1.69
General self-efficacy (mean)	0.78	0.28	0.50–1.22
Optimism (mean)	0.89	0.68	0.50–1.56
Extraversion (sum)	0.94	0.36	0.81–1.08
Neuroticism (sum)	1.98	< 0.001	1.66–2.36

*Note.* Reference categories are lower age, male gender, lower education, not working, and lower levels of general self-efficacy, optimism, extraversion, and neuroticism. Adjusted model parameters: Nagelkerke *R*^*2*^ = 0.27, Cox & Snell *R*^*2*^ = 0.08, Model χ^2^ = 134.23, *p* <  0.001. Hosmer Lemeshow: χ^2^ = 4.49, *p* = 0.81

Second, the analysis was re-run using lifetime anxiety as the outcome variable, and the results are displayed in Table 4. Echoing the results from the previous analyses, higher age reduced the odds of lifetime anxiety somewhat, whereas higher neuroticism increased the odds. In addition, the odds of reporting lifetime anxiety were reduced by having employment and by higher levels of general self-efficacy.

**Table 4:** Adjusted logistic regression analysis showing associations between the study variables and lifetime anxiety (*n* = 1684) for participants in the Norwegian population (NorPop) study, data collected 2015–2016

	Adjusted model		
Independent variables	OR	*p*	95% CI
Age increase in 10 years	0.88	< 0.01	0.80–0.96
Gender (male vs. female)	1.17	0.29	0.88–1.55
Education (≤12 yrs. vs. > 12 yrs.)	0.75	0.05	0.57–1.00
Working (no vs. yes)	0.71	< 0.05	0.53–0.95
General self-efficacy (mean)	0.75	< 0.05	0.58–0.95
Optimism (mean)	0.97	0.84	0.71–1.32
Extraversion (sum)	0.97	0.51	0.90–1.05
Neuroticism (sum)	1.74	< 0.001	1.60–1.88

*Note.* Reference categories are lower age, male gender, lower education, not working, and lower levels of general self-efficacy, optimism, extraversion, and neuroticism. Adjusted model parameters: Nagelkerke *R*^*2*^ = 0.31, Cox & Snell *R*^*2*^ = 0.20, Model χ^2^ = 361.60, *p* < 0.001. Hosmer Lemeshow: χ^2^ = 12.9, *p* = 0.11

## Discussion

This study investigated the prevalence of self-reported anxiety and associated factors in the Norwegian general population. Prevalence rates were 6.6% for current anxiety and 21.7% for lifetime anxiety, and the rates were higher for women than for men. However, the association between gender and anxiety vanished in the multivariate analysis along with most other bivariate associations. Adjusting for all variables, higher age reduced the odds of having current anxiety, whereas higher neuroticism increased the odds.

The prevalence rates of current and lifetime anxiety, as revealed in this study, indicate that self-reported anxiety may be somewhat more prevalent than rates of diagnosable disease. In comparison, lifetime prevalence rates for any anxiety disorder, at 16.6% [23] and 13.6% [8], have previously been reported. This is in line with research that has shown that self-report measures tend to yield a substantially higher frequency of cases, compared to the frequencies obtained by clinical diagnosis [24].

Higher prevalence of anxiety among women, compared to men, was confirmed in our study’s group comparisons. This reflects the uniform agreement across studies that women display higher levels of mental health problems in general [8, 10, 25]. The lack of association between gender and anxiety when adjusted for neuroticism is in line with previous research suggesting that women are more prone to anxiety because of their higher levels of neuroticism [26].

Our finding that older age groups had less anxiety is in line with another Norwegian study that found a relatively small proportion of care-dependent elderly persons (10.7%) that was considered to have psychological distress [27]. Some have suggested that the life experience associated with higher age is a resource for better coping [15], and that this experience may contribute to explain the lower prevalence of anxiety found in older persons [8]. On the other hand, Volkert and co-workers [28] suggested that low rates of social phobia in older age may be due to less exposure to demanding social situations.

More anxiety in younger age groups is a matter of concern. In fact, mental health problems appear to be increasing among young Norwegians, especially among women [16]. Recent research on Norwegian students enrolled in higher education showed high levels of mental health problems [17], and that psychological distress tended to increase through the study program [29]. Various reasons were suggested for psychological distress in young students, such as a heavy workload and problems with establishing and maintaining relationships with peers [29].

The pattern of associations was similar when restricting the outcome variable to ‘anxiety with help-seeking’, compared to the main analysis. This supports the validity of our findings. Help-seeking in two thirds of those who had anxiety is similar to that found among people with depression in the same study sample [30], and much higher than the 25% proportion of help-seekers with diagnosed anxiety disorders in a previous Norwegian study [31]. In general, help-seeking behaviors are related to illness severity and the accessibility of healthcare services, but also to perceived stigma and own attitudes [32]. In comparison to previous studies, therefore, the relatively high proportion of help-seekers among those reporting current anxiety may indicate a high burden of mental distress. Alternatively, it might indicate low stigma associated with help seeking, or a positive view of the possibility of getting appropriate professional help.

In the additional analysis using lifetime anxiety as outcome, the same pattern of associations was found. However, we also found that employment and higher levels of self-efficacy were associated with lower odds of experiencing anxiety in a lifetime perspective. Having employment and self-affirming beliefs about one’s coping abilities may buffer against mental health problems like anxiety [5, 33]. On the other hand, having anxiety problems may decrease one’s employment opportunities and decrease one’s sense of mastery and coping with life. Bandura [34] used the term ‘reciprocal causation’ to denote the interrelationships between self-efficacy and behavioral and state variables. In combination, the results also suggest that employment and general self-efficacy may be more readily associated with durable states (like lifetime anxiety) than with more fluctuating states (like current anxiety).

### Study strengths and limitations

The use of a large sample, and one that is considered fairly representative of the Norwegian population, are strengths of this study. In addition, using several personality traits as concurrent predictors of anxiety increases the trustworthiness of the results. A limitation is concerned with measuring anxiety with a single item, and the use of single-item measures are often discouraged. However, such measures have the advantage of being short, flexible, and easy to administer, and they are cost-efficient and have better face validity in comparison to multi-item scales [35]. Single-item self-report measures have also been shown to be reliable, as estimated by test–retest correlations [36] and correlations with clinical diagnosis [37]. The validity of our findings were supported by a comparison of the results derived from analyses using three different outcomes; current anxiety, current anxiety with help seeking, and lifetime anxiety. Cross-sectional studies are commonly used as the source of prevalence data. However, the cross-sectional study design precludes us from concluding about the nature of the detected associations.

## Conclusion

The point prevalence of anxiety in the Norwegian general population sample was 6.6%, whereas the lifetime prevalence was 21.7%. Current and lifetime anxiety was more prevalent among women than among men. Higher age reduced the odds of current anxiety, whereas neuroticism increased the odds.

## Abbreviations

EPQ: Eysenck Personality Questionnaire
GSE: General Self-Efficacy Scale
LOT-R: Life Orientation Test-Revised
M: Mean value
NORPOP: The Norwegian Population Study
OR: Odds ratio
SD: Standard deviation
